# Perceived barriers and facilitators to breast-feeding support practices in hospitals and birthing facilities in the USA

**DOI:** 10.1017/S1368980024002635

**Published:** 2025-01-07

**Authors:** Bee-Ah Kang, Sarah Gonzalez-Nahm, Sara E Benjamin-Neelon

**Affiliations:** 1 Department of Health, Behavior and Society, Johns Hopkins Bloomberg School of Public Health, Baltimore, MD 21205, United States; 2 Department of Nutrition, School of Public Health and Health Sciences, University of Massachusetts, Amherst, MA 01003, United States

**Keywords:** Human milk, Baby Friendly Hospital Initiative, Infant feeding, Hospital administrator, Breastmilk

## Abstract

**Objective:**

The Baby-Friendly Hospital Initiative (BFHI) designation is known to increase breast-feeding rates in the USA. However, less is known about barriers and facilitators to breast-feeding support practices in BFHI hospitals and how they differ from non-BFHI hospitals. We examined what barriers and facilitators are perceived to affect breast-feeding practices among BFHI and non-BFHI hospital administrators and further explored factors that presented challenges to the adoption and continuation of breast-feeding support practices.

**Design:**

Cross-sectional study was conducted. We measured whether hospitals were implementing 12 breast-feeding support practices and identified barriers and facilitators to the practices. The survey questionnaire included both structured and open-ended questions.

**Setting:**

This study included hospital administrators from both BFHI and non-BFHI hospitals from all regions of the USA to help elucidate potential differences.

**Participants:**

A stratified random sample of 50 % of BFHI and 50 % of non-BFHI hospitals was obtained. The final sample size included 113 BFHI and 177 non-BFHI hospital administrators.

**Results:**

Low interest among mothers was reported as the most significant barrier to providing breast-feeding support among all administrators. Non-BFHI hospital administrators were more likely to report cost, nursing staff and physician resistance and hospital infrastructure as barriers to initiating practices. In-person training was cited as the most important facilitator among both groups.

**Conclusions:**

Strengthening prenatal education for mothers and trainings for administrative and nursing staff and physicians is warranted in BFHI and non-BFHI hospitals. Staff management and hospital infrastructure need to be improved particularly in non-BFHI hospitals to provide adequate breast-feeding support for mothers.

Breast-feeding has numerous health benefits for mothers and children. It reduces maternal risk of some cancers, type 2 diabetes and hypertension and prevents immediate or long-term disease and illness among children^(1)^. At the national level, breast-feeding helps prevent premature mortality as well as economic and environmental costs^(2,3)^. The 2030 Healthy People Goals established by the U.S. Department of Health and Human Services^(4)^ stipulated two objectives to increase the proportion of infants who are breast-fed at 1 year (MICH-16) and exclusively breast-fed through 6 months (MICH-15), putting an emphasis on breast-feeding duration. Setting breast-feeding as a national priority and achieving breast-feeding duration requires timely and comprehensive engagement of and commitment from hospitals and birthing facilities because the environment in which a mother gives birth may affect breast-feeding initiation and continuation^(5)^. However, traditional practices in hospitals, including mother–infant separation and formula supplementation, set obstacles to integrating breast-feeding support practices into routine care.

To enhance maternal and child care and encourage hospitals to employ breast-feeding support practices globally, the WHO and UNICEF launched the Baby-Friendly Hospital Initiative (BFHI) in 1991^(6)^. The initiative aimed to scale up ten evidence-based practices (Table 1) for hospitals and their staff to support successful breast-feeding. Hospitals become designated as Baby-Friendly if they comply with the standards of BFHI and implement the *Ten Steps to Successful Breastfeeding* ^(8)^. Studies have demonstrated that BFHI is effective in promoting breast-feeding and health outcomes among mothers and infants^(9,10)^. A systematic review found that adherence to the BFHI Ten Steps was associated with increased likelihood of any or exclusive breast-feeding globally^(11)^. In the USA, the BFHI certification was found to be effective in increasing exclusive breast-feeding rates across various demographics^(12)^ and reducing disparities in breast-feeding outcomes^(13)^. The CDC’s Maternity Practices in Infant Nutrition and Care survey data also showed that hospitals with the BFHI designation had 13·6 % higher exclusive breast-feeding rates than hospitals without the designation^(14)^.

**Table 1.** A list of breast-feeding support practices

Have a written breast-feeding policy that is routinely communicated to all health care staff
Train health care staff in the skills necessary to provide optimal breast-feeding-friendly care and support
Inform all pregnant women about the benefits and management of breast-feeding
Help mothers initiate breast-feeding within 1 h of birth
Show mothers how to breast-feed and how to maintain lactation, even if they are separated from their infants
Give infants no food or drink other than breast-milk, unless medically indicated
Practice rooming in – allow mothers and infants to remain together 24 h a day
Encourage breast-feeding on demand
Give no pacifiers or artificial nipples to breast-feeding infants
Foster the establishment of breast-feeding support groups and refer mothers to them upon discharge from the hospital or birth center
Prohibit marketing of formula to mothers in the form of bags, samples, coupons or other materials*
Do not accept financial incentives from formula companies*

* We added two additional practices to the *Ten Steps* given the issue of accepting free infant formula and materials used for promotion efforts of formula companies among hospitals. These statements were added in compliance with the International Code of Marketing of Breast-milk Substitutes^(7)^.

The total number of BFHI-designated hospitals has substantially increased over the past decade, having more than 1 million infants born each year in BFHI hospitals in the USA.^(15)^. Although wide BFHI adoption has contributed to the overall growth in breast-feeding rates, progress in breast-feeding appears to have stagnated in recent years. Data in 2020 show that the rates of any breast-feeding (83·1 %) are lower than rates from 2015 to 2019 (83·2–84·1 %), and exclusive breast-feeding rates at 3 months and 6 months have also decreased or remained constant since 2016, remaining far below national goals^(4,16,17)^. The 2030 objective (MICH-15) of achieving 42·4 % of infants exclusively breast-fed for the first 6 months also shows negligible improvement from 2020 data (25·4 %)^(4)^. Furthermore, large geographical and racial disparities in breast-feeding initiation have persisted in the country^(18,19)^.

Improving breast-feeding support practices in hospitals has the potential to address these gaps in the national trends and disparities^(13)^. It is thus imperative to identify factors that hamper breast-feeding practices in hospitals. Prior studies revealed that barriers, including maternal exhaustion, family influence and lack of skilled hospital personnel, affect breast-feeding support practices^(20–22)^. A few qualitative studies found that breast-feeding education and interprofessional collaboration among staff helped promote breast-feeding in a hospital setting^(22,23)^. Nevertheless, there is lack of evidence on how barriers and facilitators to breast-feeding support practices vary by BFHI status, limiting our understanding of the unique needs and circumstances of BFHI and non-BFHI hospitals. Furthermore, little is known about how barriers to on-going practices differ from barriers that prevent hospitals from adopting new initiatives to support breast-feeding. A thorough investigation of factors associated with breast-feeding practice implementation may offer useful information for hospital leadership and health workers to develop strategies that are integrative yet tailored to the hospital BFHI status.

Our study aimed to (1) examine how barriers and facilitators are perceived to affect breast-feeding practices among BFHI and non-BFHI hospital administrators across the USA and (2) explore factors that present challenges to the adoption and continuation of breast-feeding support practices among hospitals.

## Methods

### Study design

We administered a cross-sectional survey to hospital administrators across the USA from fall 2019 to spring 2020 to obtain point-in-time data on facility breast-feeding practices and policies. This study was deemed exempt by the Johns Hopkins Bloomberg School of Public Health Institutional Review Board (IRB No: 00009842).

### Setting

This study included hospital administrators from both BFHI and non-BFHI hospitals from all regions of the USA to help elucidate potential differences. Recent evidence found that exclusive breast-feeding rates were higher in BFHI hospitals than non-BFHI hospitals^(24)^. Geographically, both BFHI designated and non-BFHI hospitals are equally located across regions in the USA with higher concentration in areas with high population densities. Despite recent growths in BFHI penetration, however, the percent change in increase in BFHI designation is known to be relatively lower in areas with high socio-economic disadvantage^(24)^.

### Sample

For this exploratory study, the research team mailed electronic surveys using REDCap to a stratified random sample of BFHI and non-BFHI hospitals. The sample included 50 % of BFHI hospitals and 50 % of non-BFHI hospitals. As there are fewer BFHI than non-BFHI hospitals in the USA, the sample of BFHI hospitals was smaller than the non-BFHI sample. We stratified the sample based on hospital size (i.e. the number of beds) using American Hospital Association data (2019). We categorised hospitals as small if they had one to ninety-nine beds, medium if they had 100 to 299 beds or large if they had 300 or more beds. We obtained bed size information through online searches if the American Hospital Association dataset did not include hospitals’ bed size information. We categorised standalone birthing facilities without information on bed sizes as small. We employed equal stratified sampling, where each stratum (size) of hospital was allocated the same sample size, to ensure equal representation in the sample and reduce sampling bias.

**Figure 1.**
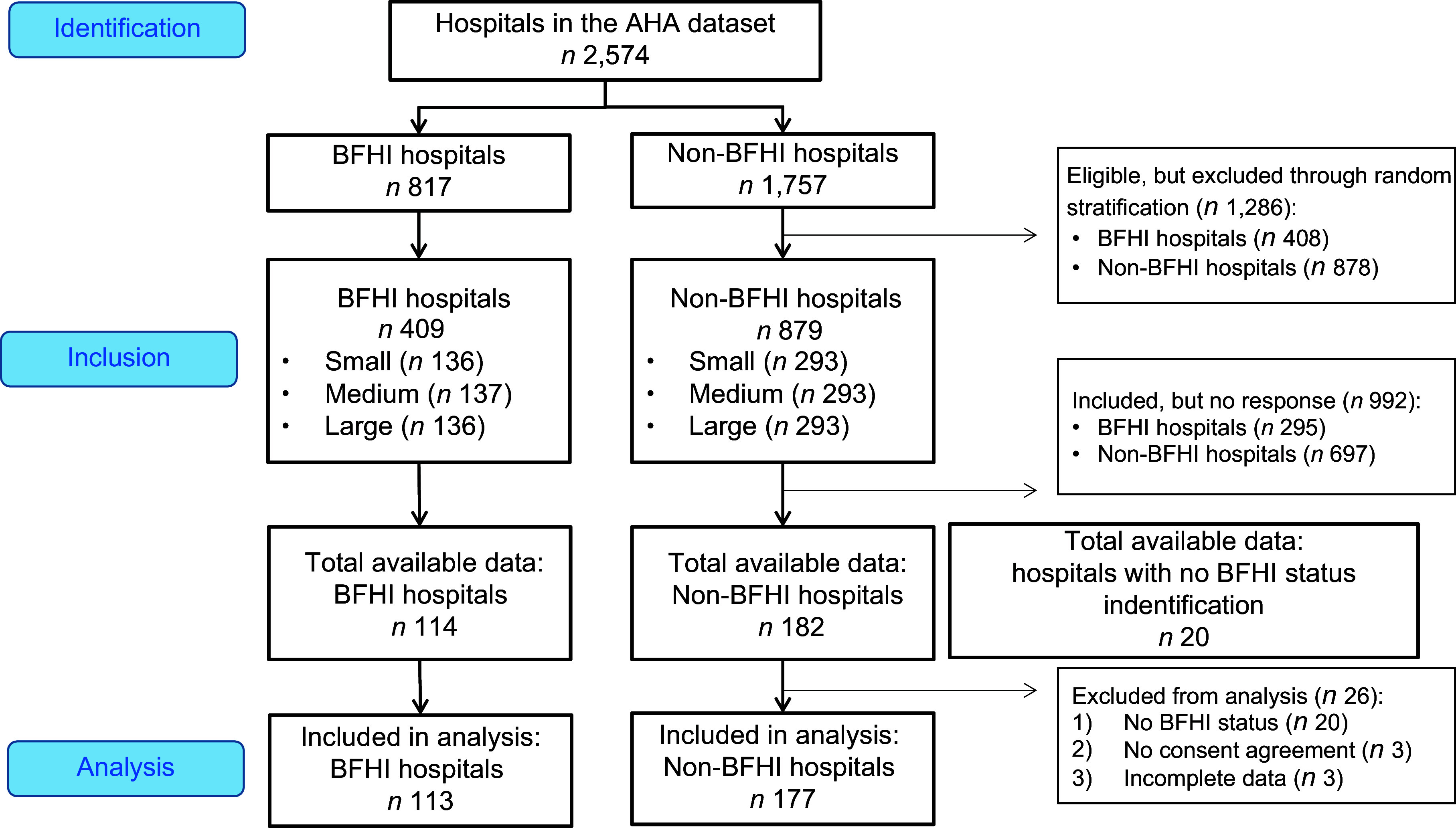
Flow chart of participants.

All hospitals listed in the American Hospital Association database were eligible to be selected. Among the 2574 hospitals in the database, there were 817 BFHI hospitals and 1757 non-BFHI hospitals. Of those, we randomly administered electronic surveys to 409 BFHI hospitals and 879 non-BFHI hospitals. After eliminating duplicates from the hospital data, we had a final sample of 1285 birthing facilities. In total, 316 hospitals completed the survey. We removed twenty-six hospitals prior to analysis because they did not provide consent or complete the survey in its entirety. The final sample size was 290 (113 BFHI and 177 non-BFHI hospitals), with adequate number for each to conduct a statistical test for comparison. The sampling procedure is described in Fig. 1 following the STROBE guidelines^(25)^.

### Measurement

To assess perceived barriers to breast-feeding support practices, we first identified hospitals’ current practices with twelve questions that entail the *Ten Steps to Successful Breastfeeding* ^(8)^(Table 1). These questions reflect the earlier version of the ten steps to capture practices based on the guidelines hospitals were likely following at the time.

If administrators indicated their hospitals were implementing any of the twelve breast-feeding support practices, we asked them to select all applicable barriers to on-going practices, using a list of nine barrier options. We coded zero for nonselected and one for selected barriers. We then asked administrators to select the most significant barrier. Subsequently, to identify factors that hinder the adoption of new practices, we asked participants to select applicable barriers for the breast-feeding support practices that are not are being implemented, using the same list of nine factors. We categorised responses into zero and one. We then asked administrators to select the most significant barrier. Additionally, we asked participants to describe additional challenges experienced in hospitals, using an open-ended question. Also, we assessed facilitators to breast-feeding support practices by asking participants to indicate resources that had helped their practices. Participants chose all applicable answers from a list of nine suggested facilitators with a binary option. We then asked participants to select the most significant facilitator from the same list. Additional facilitators experienced among participants were collected from write-in responses.

To ascertain perceived barriers and facilitators by hospital status, we asked participants to categorise their hospital’s current BFHI designation as either established BFHI, in-process (emerging) BFHI, no BFHI designation or prior BFHI designation (not renewed). We categorised established and emerging BFHI hospitals as BFHI hospitals and those that did not have or did not renew the designation as non-BFHI hospitals.

The questionnaire was developed for this study. The instrument included several demographic characteristics^(26)^ and questions about selecting the most significant barrier/facilitator^(27)^ informed by previous studies. The questionnaire was reviewed and discussed by the study team to reflect study participants and hospitals it is intended for. We integrated strategies into survey development to prevent potential biases. Our approach to capturing textual information about perceived barriers and facilitators mitigated any bias in providing predetermined options in the survey. Also, using multiple scales (i.e. multiple choices, single rank and free response) reduced potential acquiescence bias in indicating hospital experience with a list of factors.

### Data collection

We sent a letter of invitation and survey description to hospital administrators via e-mail in fall 2019. The administrators included the chief executive officer, the president or vice president or chief nursing officer. If an e-mail was not delivered and bounced back, we contacted hospitals via phone. We sent reminders each week for up to 3 weeks. The survey was designed to collect both quantitative and qualitative responses and be completed in 20 min. We provided a $20 electronic gift card upon completion of the survey. We obtained informed consent through completion of the first question of the survey. This study did not collect personally identifiable data to ensure confidentiality. Detailed methods of this study are available elsewhere^(28)^.

### Data analysis

We calculated frequencies and percentages for categorical and binary demographic characteristics of administrators and hospitals. We presented these results by BFHI status. We performed exact Pearson *χ* ^2^ tests and Fisher’s exact tests to examine differences in barriers and facilitators to breast-feeding support practices by BFHI status with a significance level of *α* < 0·05. We then used the Bonferroni correction for each analysis to provide conservative alpha values, accounting for multiple testing. Since we had nine single degree of freedom tests within each set of assessment, we adjusted the *P* values by multiplying by nine. The adjusted *P* values greater than one are considered equal to one in the correction, indicating no evidence for rejecting the null hypothesis. Also, we calculated frequencies and percentages for the most significant barriers and facilitators. We removed missing or incomplete data from analysis (*n* 26). We conducted statistical analyses using STATA 14.2 for Mac (StataCorp).

A researcher trained in qualitative research manually conducted summative content analysis^(29)^ for write-in answers by identifying and quantifying the use of certain keywords. The researcher then inductively generated categories and put quotes into themes to infer meaning from frequency counts for each theme. The other team members reviewed the categorisation of themes and selected example quotes to iteratively refine results. The team members’ mixed levels of experience in research on breast-feeding practices in US hospitals provided both internal and external perspectives during analysis and ensured rigorous interpretation of participant report. Moreover, the primary analyst blinded characteristics of participants/hospitals to mitigate biases in the interpretation of data. We conducted qualitative analysis using Excel 16.30 for Mac (MicrosoftCorp).

## Results

### Demographic characteristics

Table 2 shows administrator and hospital characteristics. Most hospital administrators were female (96·6 %). Administrators were mostly White (93·5 %), followed by Black (3·1 %) and American Indian (1·3 %). Among White administrators, 14 (5·0 %) were Hispanic/Latinx and 261 were non-Hispanic/Latinx (94·9 %). The majority of respondents (85·8 %) had completed four-year college or graduate education. Approximately one-third of administrators (34·2 %) reported having worked in their current hospitals between 1 and 5 years, and 39·7 % had worked in their hospitals over 10 years. Totally, 186 hospitals (64·4 %) were associated with a larger health system. The number of hospitals varied across regions. The South Atlantic region had the most BFHI hospitals (21·4 %), and the East North Central region had the most non-BFHI hospitals (18·2 %).

**Table 2:** Demographic characteristics of administrators and hospitals by baby-friendly hospital initiative designation

Administrator characteristics	All (*n* 290)*		BFHI (*n* 113)*		Non-BFHI (*n* 177)*	
	*n*	%	*n*	%	*n*	%
Age						
34	30	10·3	15	13·3	15	8·3
35–44	82	28·3	35	31·0	47	26·6
45–54	77	26·6	23	20·4	54	30·5
55–64	89	30·7	34	30·1	55	31·1
65	12	4·1	6	5·3	6	2·5
Gender						
Female	280	96·6	107	94·7	173	97·7
Male	10	3·4	6	5·3	4	2·3
Race						
American Indian or Alaska Native	4	1·3	2	1·8	2	1·1
Asian/Asian American	3	1·0	2	1·8	1	0·6
Black/African American	9	3·1	5	4·4	4	2·3
White or Caucasian	275	93·5	105	92·9	170	96·0
N/A†	3	1·0	0	0·0	3	1·7
Hispanic/Latinx ethnicity	15	5·2	8	7·0	7	3·9
Education						
Some college/trade school	1	0·3	0	0·0	1	0·6
Associate (two-year) degree	40	1·4	14	12·4	26	14·7
Four-year college degree	121	41·7	44	38·9	77	43·5
Graduate school degree or higher	128	44·1	55	48·7	73	41·2
Position title						
Department/program director	88	30·3	35	31·0	53	29·9
Nurse/unit manager	67	23·1	22	19·5	45	25·4
President or vice president	9	3·1	4	3·5	5	2·8
Clinical lead/supervisor	36	12·4	14	12·4	22	12·4
Executive leadership	17	5·9	10	8·8	7	4·0
Lactation care provider/nurse	57	29·7	19	16·8	38	21·5
Physician	2	0·7	2	1·8	0	0·0
Unspecified	4	1·4	4	3·5	0	0·0
Position length						
1 year	27	9·3	12	10·6	15	8·5
1–5 years	96	33·1	38	33·6	58	32·9
5–10 years	50	17·2	22	19·5	28	15·9
10 years	116	40·0	41	36·3	75	42·6

Hospital characteristics	All (*n* 290)*		BFHI (*n* 113)*		Non-BFHI (*n* 177)*	
	*n*	%	*n*	%	*n*	%
Health system association						
No	103	35·5	33	29·5	70	39·5
Yes	186	64·1	79	70·5	107	60·5
Region‡						
New England	13	4·5	6	5·4	7	4·0
Mid-Atlantic	38	13·1	17	15·2	21	11·9
East North Central	47	16·2	15	13·4	32	18·1
West North Central	39	13·4	12	10·7	27	15·3
South Atlantic	50	17·2	24	21·4	26	14·7
East South Central	19	6·6	9	8·0	10	5·6
West South Central	33	11·4	11	9·8	22	12·4
Mountain	25	8·6	5	4·5	20	11·3
Pacific	25	8·6	13	11·6	12	6·8
# of hospital beds§						
1–99	105	36·2	35	32·1	70	40·7
100–299	69	23·8	26	23·9	43	25·0
≥ 300	91	31·4	42	38·5	49	28·5
Unsure	12	4·1	6	5·5	6	3·5
N/A||	4	1·4	0	0·0	4	2·3

* Percentages not adding up to 100 are due to missing or check-all-that-apply answers.

† Prefer not to answer.

‡ New England (CT, ME, MA, NH, RI, VT); Mid-Atlantic (NJ, NY, PA); East North Central (IL, IN, MI, OH, WI); West North Central (IA, KS, MN, MO, NE, ND, SD); South Atlantic (DE, FL, GA, MD, NC, SC, VA, DC, WV); East South Central (AL, KY, MS, TN); West South Central (AR, LA, OK, TX); Mountain (AZ, CO, ID, MT, NV, NM, UT, WY) and Pacific (AK, CA, HI, OR, WA).

§ Total number of hospital beds if a birthing facility is affiliated with a hospital.

|| Birthing facility not affiliated with a hospital.

### Barriers to breast-feeding support practices

Administrators from both BFHI (*n* 22, 19·5 %) and non-BFHI hospitals (*n* 49, 27·7 %) indicated that mothers’ low interest in breast-feeding was the most significant barrier to current breast-feeding support practices in which hospitals were engaging (Table 3). Among all hospitals (BFHI and non-BFHI), competing priorities of nursing staff (*n* 136, 46·9 %), nursing staff’s resistance to change (*n* 113, 39·0 %) and physician’s resistance to change (*n* 110, 37·9 %) were most frequently reported when participants chose all applicable barriers. There were no differences by BFHI hospital status in likelihood of reporting low interest among mothers, nursing staff resistance, cost and physician resistance as barriers to current breast-feeding support practices.

**Table 3.** Perceived barriers to breast-feeding support practices by baby-friendly hospital initiative designation

	All (*n* 290)		BFHI (*n* 113)		Non-BFHI (*n* 177)			
Perceived barriers to breast-feeding support practices (being implemented)*	*n*	%†	*n*	%†	*n*	%†	*χ* ^*2*^ ‡	*P*
Cost	44	15·7	18	15·9	26	14·7	0·082	1·000
Low interest in breast-feeding among mothers	105	36·2	37	32·7	68	38·4	0·962	1·000
Nursing staff resistance to changes	113	39·0	41	36·3	72	40·7	0·560	1·000
Physician resistance to changes	110	37·9	49	43·4	61	34·5	2·320	1·000
Management-level resistance to changes	10	3·5	4	3·5	6	3·4	0·005	1·000
Competing priorities of nursing staff	136	46·9	51	45·1	85	48·0	0·231	1·000
Competing priorities of physicians	67	23·1	23	20·4	44	24·9	0·788	1·000
Management-level competing interests	18	6·2	6	5·3	12	6·8	0·256	1·000
Lack of infrastructure	68	23·5	23	20·4	45	25·4	0·988	1·000
Most significant barrier	Low interest among mothers		Low interest among mothers		Low interest among mothers		–	–
	71	24·5	22	19·5	49	27·7		

	All (*n* 290)		BFHI (*n* 113)		Non-BFHI (*n* 177)			
Perceived barriers to breast-feeding support practices (not being implemented)*	*n*	%†	*n*	%†	*n*	%†	*χ* ^*2*^ ‡	*P*
Cost	44	15·2	8	7·1	36	20·3	**9·421**	0·019
Low interest in breast-feeding among mothers	52	17·9	8	7·1	44	24·9	**14·815**	0·001
Nursing staff resistance to changes	63	21·7	11	9·7	52	29·4	**15·651**	< 0·001
Physician resistance to changes	36	12·4	6	5·3	30	17·0	**8·594**	0·030
Management-level resistance to changes	5	1·7	1	0·9	4	2·3	0·770	1·000
Competing priorities of nursing staff	48	16·6	9	8·0	39	22·0	**9·884**	0·015
Competing priorities of physicians	20	6·9	9	8·0	11	6·2	0·329	1·000
Management-level competing interests	10	3·5	1	0·9	9	5·1	3·654	0·503
Lack of infrastructure	41	14·1	7	6·2	34	19·2	**9·623**	0·017
Most significant barrier	Low interest among mothers		Low interest among mothers		Low interest among mothers		–	
	40	13·8	7	6·2	33	18·6		

* Administrators were asked to select as many or few applicable barriers from the list. They were then asked to select the most significant barrier from the same list.

† Values refer to the number and percentages of administrators who selected each respective barrier by the status of hospital.

‡ Values in bold are statistically significant at *P* < 0·05, adjusted by Bonferroni correction.

For breast-feeding support practices that were not currently being implemented, mothers’ low interest in breast-feeding was reported as the most significant barrier among BFHI (*n* 7, 6·2 %) and non-BFHI (*n* 33, 18·6 %) hospital administrators (Table 3). When participants selected all applicable barriers, nursing staff’s resistance to change (*n* 63, 21·7 %) was reported as the most prevalent barrier, followed by mothers’ low interest (*n* 52, 17·9 %). Overall, non-BFHI administrators were more likely to have perceived barriers to uninitiated practices, compared with BFHI hospital administrators. In particular, mothers’ low interest in breast-feeding, *χ* ^*2*^(1290) = 14·81; nursing staff’s resistance to change, *χ* ^*2*^(1290) = 15·65; cost, *χ* ^*2*^(1290) = 9·42 and lack of adequate infrastructure, *χ* ^*2*^(1290) = 9·62 were perceived as barriers among non-BFHI hospital administrators.

### Facilitators to breast-feeding support practices

Administrators from both BFHI (40·7 %) and non-BFHI hospitals (42·9 %) demonstrated that in-person training was most helpful for their breast-feeding practices among the list of facilitators (Table 4). When participants selected all applicable facilitators, in-person training (73·8 %), online training (54·5 %) and free education materials (44·1 %) were most frequently reported, and staffing agencies (2·0 %) were least often reported as facilitators among administrators (BFHI and non-BFHI combined). Convening a special taskforce was significantly more likely to be perceived as a facilitator among BFHI hospital administrators, *χ* ^*2*^(1290) = 14·11, compared with those in non-BFHI hospitals. No significant differences were found between BFHI and non-BFHI hospital administrators in the rest of the facilitators.

**Table 4.** Perceived facilitators to breast-feeding support practices by baby-friendly hospital initiative designation

Facilitators to breast-feeding support practices*	All (*n* 290)		BFHI (*n* 113)		Non-BFHI (*n* 177)		*χ* ^*2*^ ‡	*P*
	*n*	%†	*n*	%†	*n*	%†		
Online training	158	54·5	67	59·3	91	51·4	1·727	1·000
In-person training	214	73·8	87	77·0	127	71·8	0·979	1·000
Free training	125	43·1	41	36·3	84	47·5	3·512	0·548
Free materials	128	44·1	46	40·7	82	46·3	0·883	1·000
Lectures/grand rounds	73	25·2	35	31·0	38	21·5	3·307	0·621
Staffing agencies	6	2·1	2	1·8	4	2·3	0·082	1·000
Working with external organisations	85	29·3	39	34·5	46	26·0	2·419	1·000
Working with external consultant	103	35·5	32	28·3	71	40·1	4·189	0·366
Convening a taskforce	82	28·3	46	40·7	36	20·3	**14·110**	0·002
Most significant facilitator	In-person training		In-person training		In-person training		–	–
	122	42·1	46	40·7	76	42·9		

* Administrators were asked to select as many or few applicable facilitators from the list. They were then asked to select the most significant facilitator from the same list.

† Values refer to the number and percentages of administrators who selected each respective facilitator by the type of hospital.

‡ Values in bold are statistically significant at *P* < 0·05, adjusted by Bonferroni correction.

### Barriers and facilitators emerged from qualitative response

Table 5 provides a summary of identified themes and categories that guided qualitative data analyses. Among all participants, 118 provided narrative responses regarding perceived barriers (Table 6). Of those, thirty-four administrators provided answers unrelated to barriers (e.g. ‘None’, ‘We do practice initiation’) and were excluded from the data analysis. Qualitative responses from eighty-four hospital administrators were categorised into five themes. The most frequently reported answers were mother’s resistance, lack of awareness and socio-demographic factors.

We have a large Hispanic population, who culturally have beliefs related to colostrum and mature milk. These patients almost always request to bottle and breastfeed while in the hospital. These cultural practices make it difficult for nurses to assist these patients with successful breast-feeding while here. (Participant 16, non-BFHI, small hospital, South-Atlantic)

**Table 5:** Data analysis structure for qualitative data

Theme	Theme definition	Category	Category definition
Barriers			
Mothers’ resistance, awareness and socio-demographic factors	Any mention of maternal factors interfering with breast-feeding support practices in hospitals	Cultural and language barriers	Mothers’ resistance derived from cultural beliefs or language barriers that hinder communication with hospital staff
		Concerns about costs among low-income mothers	Low-income mothers (e.g. WIC participants) having access to free formula from other programmes or their need to go back to work without breast-feeding
		General lack of awareness or misbeliefs about breast-feeding	Mothers’ beliefs that (exclusive) breast-feeding is not important or resistance to hospital practices, including rooming in
		Lack of family support	Descriptions of lack of family support in breast-feeding or family pressure to pursue alternative feeding practices
Inadequate hospital infrastructure	Any organisational issues concerning with inadequate hospital infrastructure that hamper breast-feeding support practices	Staff shortages and management	Staff shortages or high staff turnover on the unit floor as well as inadequate staff management, including compensation and training, that limit staff’s ability to perform breast-feeding support practices
		Lack of a designated committee or taskforce	Lack of breast-feeding champions or a designated committee limiting hospital capacity in lactation support
		Lack of facilities or services within a hospital	Descriptions of inadequate supplies or room configuration needed for breast-feeding support
		Costs and funding issues	Comments about challenges concerning with costs for BFHI designation or supplies needed for breast-feeding practices.
Staff resistance or competing interests	Hospital staff’s resistance, lack of skills or interest in performing breast-feeding practices	Low interest in adhering to breast-feeding practices	Descriptions of hospital staff, including physicians, nurses and leadership, showing low interest in breast-feeding support or BFHI designation
		Lack of skills and consistency in practice	Inconsistency in breast-feeding practices among hospital staff or descriptions of current practices being not evidence based.
Social trends and external factors	Any mention of general social trends or external services that may discourage breast-feeding among mothers and hospital practices	External support or programmes that conflict with hospital practices	Descriptions of mothers’ participation in external programmes (e.g. WIC) conflicting with hospital practices or engagement of infant formula companies
		Lack of external resources or services to continue practices	Limited referrals for continuation of breast-feeding practices or lack of state/community-level resources that support breast-feeding practices in hospitals
		Low delivery rates in hospitals	Mention of low frequency of deliveries in hospital leading to challenges in ensuring optimal practices or improving skills among hospital staff
		Social trends (social media campaign)	Descriptions of general social trends, including social media campaign, conflicting with exclusive breast-feeding recommendations
Hospitals’ preference for mother-friendly practices	Hospital leadership or staff’s prioritisation of mothers’ decisions that are often counter to breast-feeding recommendations	Health concerns of mothers.	Hospitals’ prioritisation of maternal exhaustion or health conditions over practicing rooming in or early initiation of breast-feeding
		Mothers’ right to make their own decisions	Hospitals’ prioritisation of decisions made by mothers even when they are against recommendations
Facilitators			
Improving hospital infrastructure	Organisational factors that support the implementation of breast-feeding support practices, including funding, staffing and resources.	Adequate staffing and engaging lactation support providers	Engaging additional staff or lactation support providers to current staff to ensure quality of care for mothers.
		Organising a designated committee or taskforce	Organising a designated committee or taskforce in hospital to collectively develop plans for addressing barriers
		Establishing hospital policies and achieving consensus	Descriptions of the importance of having policies that are communicated across different hospital units or staff with different roles
		Securing and management of funding	Proper management of funding for BFHI designation or medical supplies needed for breast-feeding practices
		Ensuring resources within hospital to support practices.	Descriptions of hospitals equipped with resources (e.g. donor milk and milk warmer) to continue practices
Training staff and providing proper training materials	Description of staff training and provision of educational materials, as well as its connection to education for mothers	Frequent training for staff with varying modalities	Regular training required for hospital staff. Mention of the need to utilize varying modalities for training
		Providing materials for education for mothers	Providing staff with education materials that can improve the quality of counseling for mothers
		Monitoring staff performance and linkage to maternal education	Performing a chart audit to track progress of breast-feeding practices among staff and linkage to education for mothers
		Encouraging staff to obtain lactation certification	Hospital-level support for staff in obtaining lactation certification
Strengthening pre/postnatal services for mothers and family	Any mention of services or resources available for mother and family that promote breast-feeding support practices	Providing early and continued education for mothers and their family	Descriptions of the need of early and continued education for mothers and family
		Implementing different modalities for education	Employing different training modalities (e.g. video, QR code, fliers and posters) to expand mothers’ access to breast-feeding information
		Free or low-cost services for low-income mothers	Mention of integrating free or low-cost services that deliver information targeted to low-income mothers and family
		Providing tailored services and resources at discharge	Offering flexible approaches suited for mothers’ conditions (e.g. rooming in upon mothers’ acknowledgement of safety instructions) and providing resources at discharge
Managing relationships between mothers and hospital staff	Benefit of maintaining a good relationship and communication between mothers and hospital staff in breast-feeding support	Staff’s respect for mothers’ concerns	Descriptions of the need to respect mothers’ decisions to maintain good relationships and improve care for mothers
		Establishing multiple communication channels between mothers and care providers	Availability of different communication channels that mothers can contact health providers when they experience challenges in breast-feeding
		Working with mothers’ family	Mention of the importance of a partnership between staff and mothers’ family
Building partnerships with stakeholders	Any mention of the importance of working with diverse stakeholders to encourage breast-feeding practices	Building a partnership among multiple stakeholders	Descriptions of the engagement of stakeholders, including researchers, regional coalitions, clinicians and administrative staff to address barriers at multiple levels
		Utilising external programmes and services	Engagement of external services (e.g. WIC peer counselors) to continue and improve existing services

**Table 6.** Common themes of perceived barriers and facilitators from qualitative responses

Perceived barriers (*n* 86)*	*n*	%
Mothers’ resistance, awareness and socio-demographic factors†	27	31·4
Inadequate hospital infrastructure (e.g. funding, staff management and support group)†	26	30·2
Staff resistance or competing interests†	14	16·3
Social trends and external factors	9	10·5
Hospitals’ preference for mother-friendly practices	8	9·3
Others (e.g. health conditions of infants)‡	2	2·3
Perceived facilitators (*n* 136)*	*n*	%
Improving hospital infrastructure (e.g. budget, staffing and policies)†	49	36·0
Training staff and providing proper training materials†	39	28·7
Strengthening pre/postnatal services for mothers and family†	32	23·5
Managing relationships between mothers and hospital staff	7	5·1
Building partnerships with stakeholders	6	4·4
Others (e.g. attitudes)‡	3	2·2

* Answers not related to perceived barriers or facilitators were removed from the total number of respondents.

† Answers applicable to more than one theme were double coded and reported in all respective categories.

‡ Responses for ‘Others’ were not categorised into any salient themes identified.

Issues pertaining to hospital infrastructure, including staff management and funding, were also frequently reported. Some participants reported: ‘Being a Baby-Friendly hospital requires the hospital to pay for formula and pacifiers. This also requires a yearly fee, which keeps increasing’ (Participant 184, BFHI, small hospital, South-Atlantic) and ‘High turnover of staff on the floor also presents a challenge for a consistent knowledge base when lactation is not available. Nursing staff can sometimes feel too overwhelmed to provide the support needed for breast-feeding dyads’ (Participant 154, non-BFHI, large hospital, Mid-Atlantic). Also, participants stated that inconsistencies in practices and conflicting interests among health workers became barriers to breast-feeding support practices.

[Physicians] are not required to receive or to give current evidence-based information regarding the management of breastfeeding and the physiology of lactation. Also, many local pediatricians are opposed to BFHI, which only reinforces the negativity parents see on social media. (Participant 286, BFHI, small hospital, East-South-Central)

With regard to facilitators, 141 participants provided narrative responses (Table 6). Of those, twenty-two administrators provided non-applicable or unclear answers (e.g. ‘None’, ‘Still exploring’); responses from 119 hospital administrators were analyzed and subsequently categorised into five themes. The most frequently reported facilitators concerned with hospital infrastructure. One participant illustrated the effect of organising a designated team on breast-feeding within the hospital:

We have implemented our clinical practice council in January 2020 to elicit our champions to come together from all areas to review, discuss, and plan … We have already seen an increase incrementally every month for exclusive breastfeeding rates. (Participant 313, non-BFHI, large hospital, East-South-Central)

Staff training and prenatal education for mothers were also mentioned. Some participants described: ‘Many staff have attended certified breast-feeding counselor course, which have helped to increase their skills and knowledge, in addition to the 20 h of education required by baby-friendly’ (Participant 100, BFHI, medium hospital, Mid-Atlantic), and ‘We are offering breast-feeding classes weekly and hoping to capture an audience of not only for the patient but including family or any other support system they have’ (Participant 114, BFHI, medium hospital, West-South-Central).

## Discussion

In this cross-sectional study of 290 hospitals across the USA, we explored perceived barriers and facilitators to breast-feeding support practices and the difference between BFHI and non-BFHI hospitals. We found that low interest among mothers was perceived as the most significant barrier to breast-feeding practices among BFHI and non-BFHI hospital administrators. No difference was found between BFHI and non-BFHI hospitals in barriers to current practices. Non-BFHI administrators were more likely to perceive cost, nursing staff and physician resistance, competing priorities of nursing staff and lack of infrastructure as barriers to adopting new practices, compared with those in BFHI hospitals. Participants cited in-person training as the most significant facilitator.

Our results are consistent with prior evidence that maternal resistance stemming from lack of knowledge, cultural beliefs and family pressure hinder breast-feeding support practices in hospitals^(30,31)^. A review on primary care interventions suggested that BFHI accreditation alone does not increase breast-feeding rates unless system-level support is accompanied by adequate education for mothers and their families^(32)^. This suggests that strengthening prenatal education, potentially with strategies for promoting family participation, may encourage mothers to promote individual knowledge and minimise pressure from family members, in turn to comply with hospital staff’s efforts to initiate breast-feeding. In addition, our findings suggest that maternal resistance prevents non-BFHI hospitals from adopting new breast-feeding practices. We suggest improving current prenatal care programmes to address mothers’ resistance would offer an opportunity for non-BFHI hospitals to expand their breast-feeding support and care.

It is worth noting that some participants attributed maternal resistance to socio-demographic factors, particularly low income and Hispanic culture, in their narrative answers. Indeed, some stated that women enrolled in the Special Supplemental Nutrition Program for Women, Infants, and Children were more likely to refuse breast-feeding as they received financial incentives for feeding their infants formula, aligning with prior evidence on Women, Infants, and Children’s challenge in meeting breast-feeding goals^(33)^. A qualitative study suggested that many formula-feeding Women, Infants, and Children participants report feeling judged by health professionals and consequently became isolated, increasing the risk for unsafe bottle-feeding practices^(34)^. It is thus imperative to take an inclusive approach and provide targeted services for this population by limiting hospital provision of formula at discharge and coordinating available Women, Infants, and Children resources, including peer counselors and lactation support providers^(33,35)^. Meanwhile, studies found that healthcare providers often held biased assumption that African American and Hispanic women would refuse to breast-feed, leading these women to receive less lactation support and limited assistance when problems arose^(36,37)^. This indicates the possibility that our participants’ report on certain racial groups may be implicitly biased and reflected in our findings. Further research is needed to better understand the association between maternal social determinants and breast-feeding support practices among health workers.

Furthermore, proper training of nursing staff and physicians is necessary for ensuring successful initiation and continuation of breast-feeding practices. We found that resistance to changes and a lack of consistency in breast-feeding practices among nursing staff and physicians were frequently reported as barriers, similar to previous research^(38)^. Breast-feeding education in the workplace may enhance confidence among hospital staff, facilitating the overall quality of breast-feeding support^(39,40)^. Our results showed that in-person and online training, as well as free training and materials, were perceived as key facilitators to breast-feeding practices across BFHI and non-BFHI hospitals. In our qualitative data, participants additionally highlighted the role of establishing varying training modalities, ensuring consistent training and getting lactation certification in improving skills among hospital staff. Since non-BFHI hospital administrators were more likely to perceive cost as a barrier to providing breast-feeding care, health workers in non-BFHI hospitals would particularly benefit from free training programmes and materials.

Our study also found that non-BFHI hospitals are more likely to experience organisational barriers, particularly cost, lack of infrastructure and competing priorities among nursing staff. The results reveal that non-BFHI hospitals are less equipped with the systems and funding needed to provide breast-feeding support and care for mothers. Our qualitative findings complementing this result showed that a lack of lactation specialists or high staff turnover, the use of a nursery and increased annual fees for BFHI subscription were cited as common organisational barriers. Prior studies presented similar findings. An institutional ethnography of nurses described that staff shortages and policies embracing formula supplementation hindered breast-feeding care provision^(39)^, and a review of research on BFHI implementation indicated that inadequate funding, a lack of strong leadership and hospital routines interfering with breast-feeding care (e.g. 24-hour rooming-in) have also been commonly reported as obstacles to breast-feeding practices^(41)^. We recommend non-BFHI hospitals ensure policies that support improved infrastructure, including adequate room configurations, staffing and systems for training and continuing education. Since non-BFHI hospitals are less likely to have enough funds to establish proper infrastructures and resources, an organisational system to apply for funding from the government may contribute to addressing the barrier.

However, state-specific strategies may be warranted given that breast-feeding laws and programs vary by state. For example, some states have policies that are more conducive for hospitals to adhere to breast-feeding practices than other states (e.g. California mandates BFHI for acute care and special hospitals, and Florida and Alaska encourage the implementation of BFHI)^(42)^. Many states also have breast-feeding recognition programmes (e.g. the five-star program in Virginia) for hospitals without the BFHI certification. Indeed, the 2022 mPINC survey data from maternity care managers and leaders showed that some states achieved higher scores in breast-feeding practices than the national average score^(43)^. While this study collected geographical data by census regional division rather than by state, we recommend future studies investigating how the experiences of hospitals differ by state, reflecting policies on BFHI and other similar programs in place.

Our data pertaining to facilitators showed that convening a task force was more likely to be perceived as a facilitator among BFHI hospital administrators, compared with non-BFHI administrators. BFHI designation may have successfully supported hospitals in organising a committee to systematically identify and tackle problems through a multidisciplinary approach. We recommend that non-BFHI hospitals adopt similar strategies by facilitating a team of diverse stakeholders, including local breast-feeding champions, community partners, as well as clinicians, and administrators, to mitigate some of the identified challenges at the organisational level. Our qualitative data further revealed that organising an interdisciplinary committee helped increase exclusive breast-feeding rates in a non-BFHI hospital. A designated task force may be effective in developing a strategic plan outlining goals and responsibilities, implementing educational interventions and ensuring supportive policies in hospitals.

Implications from our findings may extend to hospitals worldwide. Similar to our results, a case study in Australia highlighted the importance of improving funding structures to better embed the BFHI initiative within hospitals, as limited commitment from hospital management and policy support may hinder the implementation of breast-feeding programmes^(44)^. Additionally, resistance to change among medical staff and human resource constraints, such as inadequate staffing and frequent rotation, have been recognised as common barriers to BFHI implementation in Latin American and Caribbean hospitals^(45)^. Many health facilities from low- and middle-income countries, however, may face greater challenges in implementing and sustaining BFHI, and providing breast-feeding support alone can be difficult due to limited infrastructure and resources^(46–48)^. A review of studies in Sub-Saharan Africa found that essential practices, including rooming-in, are often hindered in overcrowded facilities^(47)^. Furthermore, insufficient monitoring and high attrition of trained staff have contributed to formula feeding in countries such as Niger and Ghana^(48,49)^. Although our recommendations to strengthen staff training and management are equally relevant to resource-limited settings, measures that respond to infrastructural gaps are critical. Strategies such as standardised education and messaging for community health workers and volunteers, home-based interventions for mothers with limited access to care (e.g. those who deliver at home due to distance from health facilities) and family involvement in establishing consistent infant feeding guidelines may help foster successful breast-feeding practices.

Overall, our study provided important insights into how challenges and needs vary among hospitals, informing strategies for promoting breast-feeding support practices tailored to the BFHI status. Taking an exploratory approach, our study not only demonstrated the overall U.S. hospitals’ experiences of breast-feeding services but also offered opportunities to expand on prior evidence, including mPINC data, as to why enduring disparities in breast-feeding outcomes and breast-feeding support practices exist nationwide, calling for action to address the gaps. We believe our findings inform decision-making among hospital leadership in both types of hospitals.

### Limitations

This study has several limitations to note. First, our sample’s low response rate (24·5 %) raises the issue of generalisability. Yet, our stratified sampling ensured sufficient number of BFHI and non-BFHI hospitals across all regions of the USA. Since this was an exploratory study, we suggest future research collect a nationally representative sample of hospitals, taking account of geographical factors, to address the generalisability issue. We believe that recruiting hospitals from all states can offer vital information about how a state’s enforcement of regulations on BFHI is associated with unique challenges and opportunities in implementing breast-feeding practices in a hospital. Next, our survey was distributed to hospital leadership and administrators, whereas many of our respondents included lactation care providers. Although this yielded more holistic data on hospital practices and needs, the varying extent to which administrators enlisted the help of more specialised personnel to respond to the survey is worthy of attention. We underscore that this partly indicates a lack of mutual understanding of roles and communication between administrators and breast-feeding support staff, which calls for transparency and opportunities to collaborate across teams and units^(50)^. Future studies may merit exploring any divide between the perspectives of hospital leadership and that of other clinical workforce and how operational and administrative decisions correspond to floor-level practices. Third, as this study was conducted prior to the pandemic, we did not capture any shift in breast-feeding support practices (e.g. discontinuation of in-person lactation support) particularly between 2020 and 2021, as suggested by other studies^(51,52)^. Yet, we expect that our findings shed light on hospitals’ process of normalising and improving lactation services within facilities. Lastly, although we attempted to interpret emerging meaning from qualitative responses, our electronic survey was inherently limited in obtaining in-depth participant or hospital experiences. The use of qualitative methods, including in-depth interviews with breast-feeding practitioners, may offer a critical avenue for future researchers to reveal uninvestigated challenges and opportunities.

### Conclusions

Breast-feeding is recognised as critical health behaviour that brings numerous health benefits to mothers and infants. Although BFHI designation is known to increase breast-feeding rates among mothers, less is known about what barriers and facilitators to breast-feeding support practices remain in BFHI hospitals and how the factors differ from non-BFHI hospitals. Our study found that mothers’ low interest was perceived as the most significant barrier across hospital administrators. Non-BFHI hospitals were more likely to perceive cost, lack of infrastructure, and staff resistance as barriers to initiating breast-feeding practices. In-person training was found as the most significant facilitator among participants. Hospitals should improve prenatal education for mothers and provide regular training with varying modalities for health workers. Securing funding and hospital infrastructures is needed particularly for non-BFHI hospitals.
